# ‘Who is on your health‐care team?’ Asking individuals with heart failure about care team membership and roles

**DOI:** 10.1111/hex.12447

**Published:** 2016-02-29

**Authors:** Kori A. LaDonna, Joanna Bates, Glendon R. Tait, Allan McDougall, Valerie Schulz, Lorelei Lingard, the Heart Failure/Palliative Care Teamwork Research Group, F. Burge, S. Burnett, K. Harkness, D. Marshall, R. McKelvie, P. Strachan, D. Lowery, D. Ward, S. Smith, G. Kimel, L. Nimmon, J. Shadd, M. Arnold, S. Burns

**Affiliations:** ^1^ Centre for Education Research & InnovationSchulich School of Medicine & DentistryWestern UniversityLondonONCanada; ^2^ Department of Family PracticeFaculty of MedicineUniversity of British ColumbiaVancouverBCCanada; ^3^ Department of Psychiatry and Division of Medical EducationDalhousie UniversityHalifaxNSCanada; ^4^ Department of Anesthesia & Perioperative MedicineLondon Health Sciences CentreLondonONCanada; ^5^ Western UniversityLondonONCanada; ^6^ Department of MedicineWestern UniversityLondonONCanada

**Keywords:** chronic disease, frailty, heart failure, interprofessional teams, patient‐centred care, qualitative research

## Abstract

**Background:**

Complex, chronically ill patients require interprofessional teams to address their multiple health needs; heart failure (HF) is an iconic example of this growing problem. While patients are the common denominator in interprofessional care teams, patients have not explicitly informed our understanding of team composition and function. Their perspectives are crucial for improving quality, patient‐centred care.

**Objectives:**

To explore how individuals with HF conceptualize their care team, and perceive team members' roles.

**Setting and Participants:**

Individuals with advanced HF were recruited from five cities in three Canadian provinces.

**Design:**

Individuals were asked to identify their HF care team during semi‐structured interviews. Team members' titles and roles, quotes pertaining to team composition and function, and frailty criteria were extracted and analysed using descriptive statistics and content analysis.

**Results:**

A total of 62 individuals with HF identified 2–19 team members. Caregivers, nurses, family physicians and cardiologists were frequently identified; teams also included dentists, foot care specialists, drivers, housekeepers and spiritual advisors. Most individuals met frailty criteria and described participating in self‐management.

**Discussion:**

Individuals with HF perceived being active participants, not passive recipients, of care. They identified teams that were larger and more diverse than traditional biomedical conceptualizations. However, the nature and importance of team members' roles varied according to needs, relationships and context. Patients' degree of agency was negotiated within this context, causing multiple, sometimes conflicting, responses.

**Conclusion:**

Ignoring the patient's role on the care team may contribute to fragmented care. However, understanding the team through the patient's lens – and collaborating meaningfully among identified team members – may improve health‐care delivery.

## Problem statement

Many in Canada's ageing population are affected by multiple chronic illnesses.^1^, ^2^, ^3^ Because these patients require a host of providers to address their multiple health concerns, interprofessional health‐care teams have become the preferred mode of care delivery.^1^, ^4^ However, our definition and understanding of such teams are limited, deriving largely from the health‐care professional perspective.^5^, ^6^, ^7^ While this perspective is valuable, we also need to know who *patients* perceive as their care team and how they understand their team to function. This knowledge is particularly critical if we are to meet the increasing expectation that patients engage meaningfully in their care to assist with achieving desired health outcomes.^8^

Researchers suggest that complex patients ‘reap the benefits of more eyes and ears, the insights of different bodies of knowledge, and a wider range of skills.'^4^ An interprofessional or multidisciplinary team – conceptualized in the literature as a group of clinicians who communicate about, and participate in, the care of a patient – is considered the most successful approach to chronic disease management.^4^ Also central to chronic disease management is a patient‐centred care approach, which seeks to develop common ground with patients for integrated management of their disease in a way that responds to their individual problems, fears and needs, while maximizing health promotion.^9^ Patient‐centred care can increase patient satisfaction and engagement, reduce anxiety and improve quality of life, and increase doctor satisfaction.^10^, ^11^, ^12^

Teamwork and patient‐centredness are both recognized as important for effective care of complex patients; however, patients are not usually conceptualized as a *member* of the health‐care team.^5^ Research has shown that patients are aware of health‐care teamwork, and patient satisfaction with team practices has been recognized as a valuable measure of team performance.^13^, ^14^, ^15^, ^16^ However, it is rare for patients to be asked who they perceive their care team to be. While researchers have asked patients to identify their primary care physician or a particular specialist, no studies have explored patients' perceptions of the membership and roles of their entire care team.^17^, ^18^, ^19^ With the recognition that health‐care team members are often changing, distributed across time and space, and attending to different aspects of the patient's needs, the single factor binding the team together is the patient him/herself. It makes sense, therefore to explore this critical vantage point by asking how patients define their care team.

We explore this question in the context of a larger study investigating care practices for heart failure (HF). Characterized by fluctuating symptoms, prognostic uncertainty and multiple comorbidities, heart failure is a representative case of chronic, life‐limiting illness.^20^, ^21^ The 500 000 Canadians living with HF^22^ typically receive care from numerous providers in various settings. HF care can be fractured and insufficient, making this a valuable, even iconic, setting in which to explore the question of how complex patients define their care team.^23^ Little is known about how individuals with HF perceive their team‐based care.^7^ With the recognized importance of patient self‐management in HF, it is critically necessary to understand how individuals with HF understand and participate in their care team.^24^, ^25^, ^26^ This paper explores the questions: Who do individuals with HF perceive as being on their care team, and how do they perceive their own and others' team roles?

## Methods

We used an innovative methodological team sampling unit (TSU) approach that explores how health‐care teams are defined and experienced by individuals with HF.^27^ Five Canadian cities in three provinces with differing health‐care organization for primary and secondary care and for the care of heart failure patients were identified as recruitment sites (Table 1).

**Table 1:** Description of study sites

CHF centre	Clinic size	Dedicated team members	Funding
Site 1	20–25 patients assessed weekly	NP‐run clinic: 1 FTE cardiologist (all other cardiologists can have patients followed by CNPs), 9 cardiology fellows, 4 CNPs	No dedicated provincial funding
Site 2	20–30 patients assessed weekly	2.5 FTE HF cardiologists, 1.5 FTE cardiology trainees, 1.6 FTE RN clinical nurse practitioners (CNPs), 0.6 FTE RN	No dedicated provincial funding; local opportunistic funds support 0.6 FTE CNP
Site 3	30–35 pts assessed weekly	2 FTE cardiologists, 1 FTE NP, 1.5 FTE RNs. Collaboration with local palliative care specialists who provide special access to regional programs	Hospital funded
Site 4	50–60 patients assessed weekly	4 FTE cardiologists, 2 FTE CNPs, 3 psychiatrists sharing Cardiac Psychiatry clinic, 1 internist doing palliative care module	Funding by province, and hospital
Site 5	40–50 patients assessed weekly	4 cardiologists. 1 geriatrician. 1 FTE NP. 1.2 FTE divided between two RNs	Hospital funded

Patients with advanced HF, NYHA Class III or IV (Table 2) were recruited primarily from Heart Function Clinics using a combination of convenience and purposive sampling.^28^, ^29^ We also attempted to recruit through family physician offices with limited success. A research associate (RA) at each site consulted clinic staff to identify eligible participants. At their next clinic visit, clinic staff provided eligible participants with an information sheet and informed them that a RA was available to discuss the study with them. The RA followed up with participants to answer questions, gauge interest and schedule interviews with willing patients. Four trained interviewers not involved in participants' health care conducted the interviews. Interviewers asked participants to tell the story of their HF care journey and to identify their care team members in response to the questions, ‘Who is on your health care team? Who helps you with your HF care?’ To honour our patient‐centred methodological approach, interviewers acknowledged the patient participant's role as a team member, but did not otherwise define ‘care team’ in order that participants would report their own conceptualizations. If an individual asked for clarification, interviewers explained that a ‘care team member’ was someone who provided some degree of recurring, HF‐related care for them. When an individual identified at least two other team members, and those team members consented to be interviewed, those 3+ transcripts were connected for data analysis in a team sampling unit (TSU).

**Table 2:** Index patients: demographics

	Index patients	Sex (%)		Age range	Followed by HF clinic (%)	Palliative care consult (%)	Deaths during study period (%)
ALL sites	62	Male	45 (73)	39–93	49 (79)	5 (8)	7 (11)
		Female	17 (27)				
Site 1	19	Male	13 (68)	49–91	18 (95)	0 (0)	3 (16)
		Female	6 (32)				
Site 2	12	Male	8 (67)	52–92	6 (50)	0 (0)	0 (0)
		Female	4 (33)				
Site 3	11	Male	8 (73)	39–93	9 (82)	1 (9)	4 (36)
		Female	3 (27)				
Site 4	12	Male	9 (75)	46–91	9 (75)	4 (33)	0 (0)
		Female	3 (25)				
Site 5	8	Male	7 (88)	54–84	7 (88)	0 (0)	0 (0)
		Female	1 (12)				

To determine how individuals with HF identified their team, two researchers (i) read each patient transcript and (ii) documented each person discussed by the patient as a member of his or her care team, (iii) recorded the stated title or activity of each of these individuals, and (iv) extracted patients’ qualitative descriptions of the roles of described team members, including their own. To triangulate the qualitative descriptions of team roles that were provided by individuals with HF, two researchers reviewed other transcripts in the TSU for additional role descriptions**.** Each TSU was also reviewed to identify index patients’ comorbidities. Because frailty has been identified as an important factor in this group of patients, it was assessed using the Fried phenotype.^30^, ^31^, ^32^

A multidisciplinary group of researchers – including social scientists, family physicians, a psychiatrist and palliative care specialists – used a mixed‐methods approach to analyse the data**.** Descriptive statistics were used to analyse the composition of patient‐identified care teams. Comorbidity and frailty data were also analysed using descriptive statistics. Transcripts were content‐analysed for recurring thematic patterns in patients’ perceptions of team members’ roles; data exploring the perspectives of other team members have been reported elsewhere.^33^ Excel and Nvivo10, a qualitative research software program, were used to organize and manage the data. This study was approved by the Research Ethics Board at each participating site. Each participant was assigned a pseudonym and a code representing their formal role. Data excerpts are referenced according to participant pseudonym, participant role, index patient pseudonym (to demonstrate relationships, if applicable) and site number (e.g. Site 1: Dr. Isadora, Ida's Family Physician).

## Results

### Who were the patient participants?

**Figure 1:**
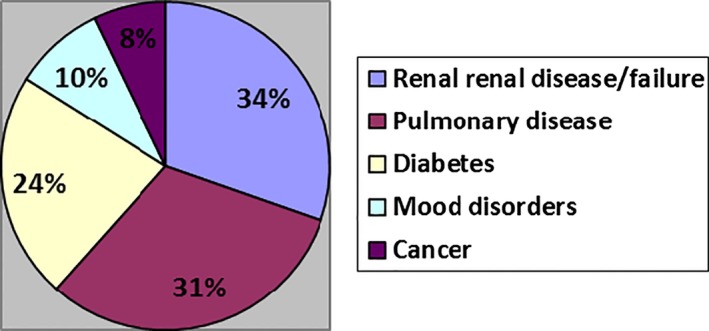
Percentage of patients who reported various comorbidities. *Some patients reported multiple comorbidities.

Sixty‐two individuals with NYHA III or IV heart failure participated (Table 2). They ranged in age from 39–93, and 27% were female (*n* = 17) and largely lived in cities (42%, *n* = 26), with fewer from smaller towns (18%, *n* = 11) and rural areas (6%, *n* = 4). 97% (*n* = 60) reported 1–7 comorbidities (mode = 1) (Figure 1); of these, the most frequently occurring were as follows: renal disease/failure (*n* = 21, 35%), pulmonary disorder, disease or dysfunction (*n* = 19, 32%), diabetes (*n* = 15, 25%), mood disorders (*n* = 6, 10%) and cancer (*n* = 5, 8%). Twenty‐six (42%) patients met all criteria of Fried et al's frailty phenotype; 14 (23%) met three and 3 (5%) patients met two.^32^ Of these, 42 (68%) patients reported exhaustion, slow walking speed or decreased physical activity; 30 patients reported weakness (48%). A total of 49 patients (79%) were seen in a Heart Function Clinic, and 5 (8%) had received a formal palliative care consult. Seven (11%) patients died during the data collection phase of the study.

### Who did individuals with HF identify as their care team?

Each patient participant identified 2–19 team members, including health professionals and informal care providers (Table 3). Most patients identified at least one family or friend caregiver (*n* = 56, 90%), nurse (*n* = 55, 89%), family physician (*n* = 55, 89%) or cardiologist (*n* = 53, 85%); however, patients commonly identified multiple members of each group. While allied health professionals and non‐cardiac medical specialists were less commonly named, 39 teams (63%) included a cardiac surgeon, nephrologist, respirologist or palliative care physician, and 20 (32%) patients considered a pharmacist, dietician, physical therapist, podiatrist or dentist part of their care team. Many patients considered themselves integral members of the care team, not passive recipients of care:

**Table 3:** Patient‐identified health‐care team members

Team members	Index patients (*n* = 62) (%)
Caregivers	56/62 (90)
Nurses	55/62 (89)
Family physicians	55/62 (89)
Cardiologists	53/62 (85)
Nephrologists	13/62 (21)
Palliative care specialists	5/62 (8)
Other specialists	20/62 (32)
Pharmacists	8/62 (13)
Other allied health professionals	12/62 (19)

I make sure and tell them everything that's been happening with me and I want them to know right down to the last detail, if I had diarrhea from pills. I'll tell them everything just so they'll know how it's affecting my body and I want them to know that. And that's my role, to make sure that they know everything that's going on with me … (Site 2: David, Index Patient).

**Figure 2:**
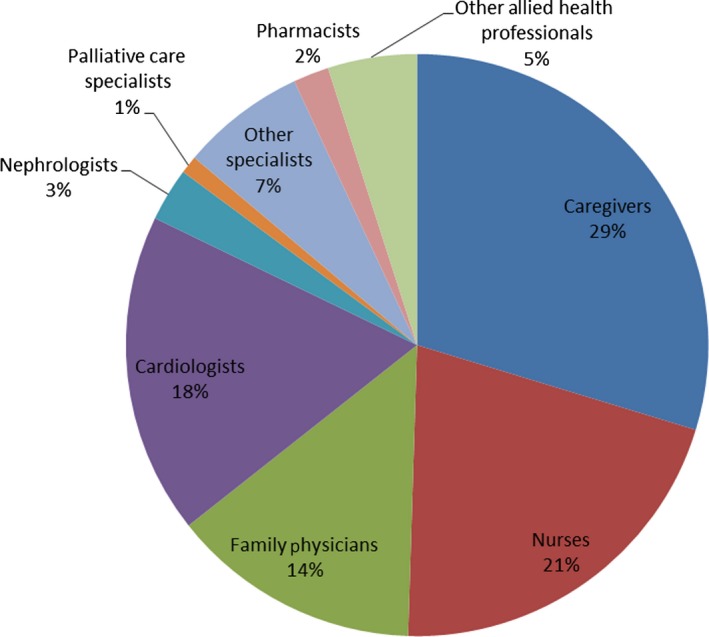
Team composition: relative frequency of team members identified by patients. *Some care team members were identified by, and formed part of a team sampling unit (TSU) of, more than one patient (e.g. 56 nurses were identified by 89 patients times).

**Figure 3:**
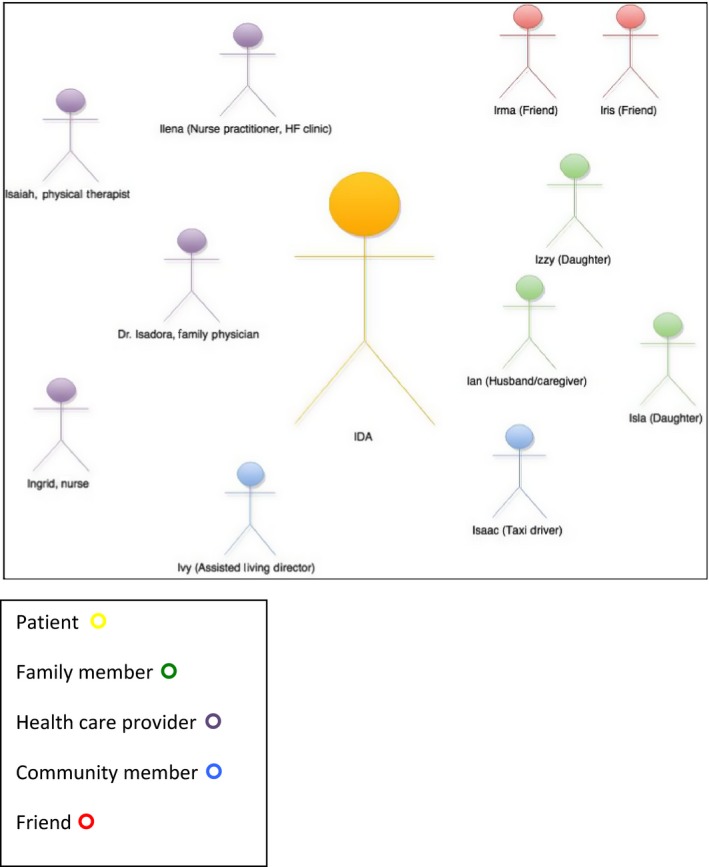
Ida's team (site 1).

The most frequently identified team members (Figure 2) were nurses (*n* = 89) and family or friend caregivers (*n* = 129); patients identified between 1 and 5 such caregivers. Also commonly identified were paid or unpaid supportive community members including clinic staff, housekeepers, church congregants and spiritual advisors. Figure 3 illustrates the members identified by Ida, a patient from Site 1.

There were site‐based differences in patients’ descriptions of their care team. The number of team members identified at each site was similar; however, the composition of the teams varied by site (Table 4). 100% of patients in Sites, 3, 4 and 5 identified nurses compared to 79% in Site 1 and 75% in Site 2; patients in Site 1 and Site 4 identified a broader range of team members, namely caregivers and community members. Of the 129 family or friend caregivers or supportive community members identified across the sites (Table 3), 80 (62%) were identified by patients in Site 1 and Site 4. Moreover, in contrast to 67% in Site 2, 100% of patients in Site 1 identified family physicians, and 42% of index patients in Site 4 identified allied health professionals while none were identified at Sites 2 or 3. Overall, approximately 90% of patients identified a family physician, nurse or caregiver/supportive community member (Table 1). The 6 patients who identified palliative care specialists all came from sites that have formal HF palliative care resources (Sites 3, 4 and 5).

**Table 4:** Site comparison of team members identified by patients (*n* = 62)

Team members	Site 1 (*n* = 19) (%)	Site 2 (*n* = 12) (%)	Site 3 (*n* = 11) (%)	Site 4 (*n* = 12) (%)	Site 5 (*n* = 8) (%)
Caregivers	18/19 (95)	10/12 (83)	10/11 (91)	11/12 (92)	7/8 (88)
Nurses	15/19 (79)	9/12 (75)	11/11 (100)	12/12 (100)	8/8 (100)
Family physicians	19/19 (100)	8/12 (67)	9/11 (82)	11/12 (92)	8/8 (100)
Cardiologists	16/19 (84)	11/12 (92)	10/11 (91)	8/12 (67)	8/8 (100)
Nephrologists	2/19 (11)	3/12 (25)	2/11 (18)	4/12 (33)	2/8 (25)
Palliative care specialists	0/19 (0)	0/12 (0)	1/11 (9)	4/12 (33)	1/8 (13)
Other specialists	7/19 (37)	2/12 (17)	5/11 (46)	5/12 (42)	1/8 (13)
Pharmacists	1/19 (5)	0/12 (0)	2/11 (18)	3/12 (25)	2/8 (25)
Other allied health professionals	5/19 (42)	0/12 (0)	0/11 (0)	5/12 (42)	2/8 (25)

### How did individuals with HF conceptualize team members’ roles?

Patients’ perceptions of the nature and importance of other team members’ roles varied according to their current needs, their relationships with providers and the structure of their local health‐care context. For some participants, the family doctor was the ‘pivot’ who directed all aspects of care:

… the reports all come back to him. He is my pivot. I'll go and have a test somewhere and I may not hear from the specialist but because (*family physician)* sees me, even sometimes once a month or once every two months, I'm constantly informed of the results of the test …. So I think he's excellent at coordinating what the others are doing (Site 2: Edward, Index Patient).

For others, specialists were perceived as the central team members. For example, in a TSU with a patient–family physician dyad with a long‐standing therapeutic relationship, the family physician spoke at length about the care and support he had provided the patient over 40 years. However, the patient positively but sparingly referenced his family physician during his interview, instead devoting time and detail to discussing the care he received at speciality clinics.

All the blood work and all that goes to him (*family physician)* and he's assigned, like I'm on Warfarin and he monitors that and my thyroid he monitors that, so the special areas that they give to the GP and the rest of the heart. Now the dialysis, they've sort of taken over. I don't have to go see three or four doctors a week. That's kind of hard. Now they've organized it so that the kidney clinic, (*hospital)* is looking after me and they give all the information to the other doctors. They're coordinated. (Site 4: Victor, Index Patient)

As this example suggests, the patient's sense of team member roles could diverge from other team members’ perceptions. Here, Victor perceived that the nephrologists have ‘taken over’ the primary role of coordinating his needs and health information.

Almost all patients described in detail the importance of family or friend caregivers to their ability to manage at home and navigate through the health‐care system. Many individuals with HF also identified as essential health professionals who do not provide ‘direct’ HF care. For example, one patient identified that she relied on her physiotherapist to help her with her mobility (Site 1: Ida, Index Patient), and another described that his dentist improved his health by managing his chronic orofacial pain (Site 1: Nicholas, Index Patient). Individuals who relied on clergy or friends for spiritual guidance talked expressively about their important roles. Finally, a number of patients included in their identified care team community members who were hired to assist with household chores or transportation. These roles were portrayed as essential by patients: without their services, participants felt that they would be unable to maintain their independence and keep up with their health‐care appointments. Ida, for instance, reported that her ‘main caregiver travelling‐wise is Isaac who is sort of a taxi driver … He looks after us like a son … And his charges are very reasonable, but it's the service he gives that's so important …. If we're going to be just a short time, he'll wait with us’ (Site 1: Ida; Index Patient). Isaac's services extended beyond driving Ida and her husband to medical appointments and included running errands, helping with household tasks, and doing their Christmas shopping Isaac perceived that his role was fundamental for providing companionship to homebound seniors: ‘… the majority of them *(customers)* always want to talk because they may be in their rooms by themselves, especially if it's a single person … most of them sort of want to chit chat with you’. Not uncommonly, Isaac's clients shared their health information with him and sought his advice, but:

I don't get involved with that because I can't comment on it properly because I'm not an expert on their area of problems. Sometimes they'll ask … I had one lady, what kind of pain medication do you take? … You should always consult with your doctors. So you don't want to get into the medical side of anything.

### How did team members perceive the patient's role on the team?

In general, patients described their role in the care team as active and central. They perceived themselves as primarily responsible for preventing symptom exacerbations and hospitalizations, with support from other team members: ‘yes, the Heart Function Clinic is there; however, they can't do everything for me. You need a team, you need to make it work for yourself…I choose to take the tools they have given me and use them as best as I can…’ (Site 3: Ophelia, Index Patient). Most individuals with HF described themselves, like Ophelia, not as being cared for *by* their team, but as having a primary role in the *functioning of* that team. They commonly characterized a core feature of their role as being proactive and in control, which they achieved largely by learning about the aetiology and symptoms of HF, following treatment recommendations, monitoring their weight, and restricting salt and fluid intake. As Leon put it, ‘I have to have control of all my health issues’ (Site 1: Leon, Index Patient).

Patients’ perceptions of their role could influence others’ perceptions. On many TSUs, the patient's HF knowledge and self‐care practices were taken as a signal that they warranted a more active role on the team. Such patients had the ability to direct the team's actions regarding their care needs:

Sylvan is pretty good, and that was the same with Farida, very good at self‐managing their heart failure. So, they would adjust their own diuretic needs, based on their symptoms or their weight. They were very good at managing their own symptoms. They would call the clinic and you would know, when they called the clinic, they were sick (Site 1: Barbara, Sylvan and Farida's Nurse).

When patients were perceived as being attuned to their symptoms, they could gain the authority to activate care processes as needed rather than following an externally dictated appointment schedule. Similarly, patients with sound awareness about their condition were given latitude to manage their health or direct their care:

He had a sense of when things were okay, and he had a sense of when things were getting out of control. He had that, and that's what I'm alluding to in his mechanical ability. He had awesome sense. He could just see it and say that works. Similarly with his body, he could push it to the extreme limit, and then when he was there, he had the good sense to say I need help. (Site 1: Dr. Akamura, Albert's Family Physician).

As this example suggests, participants with acknowledged personal or professional knowledge could play an influential role on their team. Ida (Site 1, Index Patient) had a nursing background and could understand complex medical information. As a result, her health‐care providers trusted her to synthesize their advice and use her knowledge and experience to make decisions. In particular, Ida's nurse practitioner waited for her to initiate medical appointments based on her perception of need, and her family physician trusted her to understand the implications of refusing medical interventions: ‘she was a nurse, so when I talk to her about her developing the atrial fibrillation, she's fully aware that she could have a stroke, and she knows exactly what that means …. Then we told her about Warfarin, well, she did not want to go get blood tests regularly, that was just going to be a major inconvenience to her …. she doesn't want them poking at her’ (Site 1: Dr. Isadora, Ida's Family Physician). Ida's choices were respected and shaped the team's behaviour, because she was seen by others as capable of understanding her situation.

The above examples illustrate that some patients experienced agency in their role on their care team; they were able to exert influence on the team's actions and decisions because other team members perceived that influence as legitimate due to characteristics such as strong HF knowledge and keen self‐awareness. However, other patients and team members described situations in which individuals with HF were not accorded influential roles; in fact, a number of participants described situations in which a patient's assertion of agency as a member of their care team was resisted. When individuals withheld health information from family members (Site 2: David, Index Patient), refused to comply with institutional regulations (Site 4: Carina, Index Patient), declined assistance with activities of daily living (Site 1: Bernard, Index Patient) and disregarded their restricted diet (Site 1: Albert, Index Patient), these actions could be interpreted by other team members as non‐compliance rather than as appropriate assertions of agency. A cardiologist describing his patient, Tatsumi (Site 4), who was a candidate for revascularization surgery but chose to delay making a decision about having the procedure, characterized him as:

non‐compliant in a few aspects which a lot of patients are. He still has some smoking issues. There's a lot of push and pull between him and family members or family members want him to be 100% compliant and he kind of resists 100% compliance level, I believe. He generally does what we ask him, medication wise and diet wise, but there are a few aspects where he may not be in total compliance with what we would like for him medically. (Site 1: Dr. Fabian, Farida's Cardiologist)

## Discussion

The definition of ‘my HF care team’ in this study varied by patient and, to some degree, by setting, suggesting that HF clinicians should clarify the patient's sense of who is on their team and what roles those individuals play. Some individuals with HF described small care teams consisting of a caregiver, their family physician and their HF nurse or cardiologist. Others described larger care teams made up of medical specialists such as cardiologists, family physicians, respirologists, nephrologists and electrophysiologists. Many participants reported that their key team members included allied health professionals, some conventionally associated with HF care – HF nurses, dietitians, social workers – and others not formally considered part of the HF care team, such as dentists, foot care specialists, pharmacists or physical therapists. Some participants explained that community members such as ministers or drivers were essential to their HF care.

The varied nature of 62 patients’ identified care teams is striking and suggests that these individuals, living with advanced HF and often with other chronic comorbid conditions, have their health needs served by a rich variety of individuals drawn from their family, community and health service. Policy documents depict a more narrowly defined HF care team than our data suggest: for instance, the CCS 2008 Guidelines describe the ‘heart failure team’ as consisting of heart failure specialists (cardiologists, internists or nurses) who collaborate with primary care physicians.^34^

The variations in team structure reported by our participants are influenced by both patient characteristics and local context. Patient preferences matter – a patient with strong spiritual beliefs may identify a spiritual leader as a team member, while another patient may not – but they are not the sole influence. Site‐based patterns influence team structure as well. Patients in Sites 1 and 4 identified a greater range and number of community individuals than patients in other sites, which may signal stronger community networks in these settings. Only patients in sites with funded, integrated palliative care services identified palliative care specialists as team members. And patients who attended nurse‐led HF clinics were more likely to identify a HF nurse than a cardiologist as a member of their team.

These findings suggest that we need a broader conceptualization of the HF care team, inclusive of unexpected or unconventional members who, according to patients, can play important roles in their care. The HF literature has recognized the importance of spouses and partners as ‘informal caregivers’ in HF care, particularly in relation to patients’ ability to effectively enact self‐care recommendations.^25^, ^26^, ^35^, ^36^ Our results add to this the suggestion that the emerging conceptualization of ‘informal caregivers’ might be usefully broadened to include neighbours, community members such as ministers, and informal supporters such as drivers.^37^ While not all patients may identify such a broad range of individuals, for those who do, recognition of their roles may assist health professionals to understand and collaborate with patients’ entire supportive care networks. Our previous work has suggested that such networks may function in adaptive ways not entirely predictable by conventional heart failure care algorithms.^33^ As Knowles has argued, the ‘hidden carers’ in these networks have crucial roles to play in mitigating the illness experience for complex patients; therefore, the better we understand each patient's system of hidden carers, the more effectively we can engage their abilities and relationships with patients towards maximizing patient health.^38^ Furthermore, with the growing recognition that patient self‐care practices are highly context‐dependent, a more nuanced conceptualization of HF care teams may produce important insights into how patients understand and leverage their ‘social ties’ in their daily efforts to manage their disease.^35^, ^39^, ^40^

Patients in our study perceived themselves as playing an important role on their own care team. Most characterized themselves as *active agents in* rather than *passive recipients of* their care, even if they met criteria for frailty. While other team members described many examples in which patients were trusted as autonomous team members, they also reported situations in which patient agency, characterized as ‘non‐compliance’, was resisted by the team. These results suggest that patient agency is not an individual trait but is, rather, a negotiation among team members.^41^

Social constructions of agency approach a patient's agency as not his or her own, but co‐constructed and negotiated with others to achieve specific goals.^42^, ^43^ A change in goals –.for example from aggressive to palliative care – may change the agency of the patient; it may also change the agency of other team members**.** 42 Alignment with a communally held goal, such as avoiding hospital admission, increases the individual agency of any team member. Similarly, our results suggest that when patients align with or possess health‐care knowledge and skills, their agency is increased.

In discussions of non‐compliance, team members discussed patients more as the focus of their work than as legitimate team members. This resonates with the literature on cardiovascular teams that defines teams as individuals working together to solve patient problems rather than working *with patients* to negotiate problem‐solving.^26^ The distinction suggests a nuanced difference between a philosophy of patient‐centredness, which many health professional participants invoked in their discussions, and a philosophy of patient agency as a bona fide team member. This distinction was most evident in cases in which the patient *might have been* characterized as non‐compliant but was instead understood as an active agent on the team. For instance, Dr. Akamura could have characterized Albert as non‐compliant, but instead he described him as someone who ‘would always initially comply, but when compliance ran in the way of life, he just went with life … and said I'm going to live it to the best of my ability. When I get too tight, I'll go see the doctors, and that's what he did. … and for 15 years it was great’.

As Albert's situation suggests, ‘patient agency’ is at least partly in the eye of the beholder. And this perception matters for effective teamwork. With growing awareness that patients who are active and effective participants in their own health and health‐care experience better outcomes, patient ‘activation’ or engagement has become a focus of health‐care reform efforts for complex chronic care.^44^, ^45^, ^46^ Our results complicate this notion of ‘activation’ by suggesting that it may include actions conventionally perceived by health‐care providers as ‘noncompliant’. These results resonate with recent research showing that patients with chronic illness may simultaneously experience active engagement and powerlessness in their care, and that this may be compounded when health professionals interpret as ‘noncompliance’ their efforts to cope and achieve self‐determination.^47^ Following from this, we argue that patient agency is negotiated in the context of the care team; patients’ exertions of agency can be recognized, ignored, embraced or refused by the other members of the care team. With the diversity of teams described by our patient participants, patients may experience multiple, perhaps conflicting, responses to their agency.

### Limitations

Qualitative research is local and contextual; findings are not generalizable to different clinical settings, but may be transferrable. That is, findings may resonate with clinicians, allowing explorations of their application in new contexts of practice.^48^, ^49^

Our participants were English‐speaking and recruited predominantly from urban centres. Future research could use a multilingual study design inclusive of rural centres, to explore how these factors influence team composition.

Our interest in quantifying the size and diversity of patient‐identified care teams emerged during the analysis of semi‐structured interview transcripts; therefore, not all patients were systematically surveyed regarding demographics and team characteristics. While we were able to richly describe most patients and their teams based on interview responses, some gaps remain in our demographic data.

## Conclusion

Asking individuals with HF ‘who is on your team?’ revealed a broader sense of care team membership than the traditional definition used by HF care providers. Many patients named community members as important, and most patients perceived themselves as active team members. Our findings regarding team diversity and patient agency have significant implications for health‐care efforts to improve the experiences of patients with complex, chronic disease. For such patients, it may be beneficial to ask who they consider to be on their team and to engage these individuals meaningfully in collaboration.

## Declaration of conflicting interests

The authors declared no potential conflict of interests with respect to the research, authorship and/or publication of this article.

## Funding

The authors received financial support for the research of this article from the Canadian Institutes for Health Research (CIHR) and the American Medical Association of Southwestern Ontario (AMOSO).

## Appendix 1

### Heart Failure/Palliative Care Teamwork Research Group

F. Burge, S. Burnett, K. Harkness, D. Marshall, R. McKelvie, P. Strachan, D. Lowery, D. Ward, S. Smith, G. Kimel, L. Nimmon, J. Shadd, M. Arnold, S. Burns.

## References

[1] Canadian Institute for Health Information (CIHI) . Health Care in Canada, 2011: A Focus on Seniors and Aging, 2011 Available at: https://secure.cihi.ca/free_products/HCIC_2011_seniors_report_en.pdf, accessed 15 June 2015.

[2] McDaniel S . Canada's Aging Population, 1986 Available at: http://publications.gc.ca/collections/Collection/H39-608-2002E.pdf, accessed 15 June 2015.

[3] Hoover M , Rotermann M , Sanmartin C , Bernier J . Validation of an index to estimate the prevalence of frailty among community‐dwelling seniors. Health Reports, 2013; 24: 10–17. 24258362

[4] Wagner EH . The role of patient care teams in chronic disease management. British Medical Journal, 2000; 320: 569–572. 10688568 10.1136/bmj.320.7234.569 PMC1117605

[5] Xyrichis A , Ream E . Teamwork: a concept analysis. Journal of Advanced Nursing, 2008; 61: 232–241. 18186914 10.1111/j.1365-2648.2007.04496.x

[6] Given B , Simmons S . The interdisciplinary health‐care team: fact or fiction? Nursing Forum, 1977; 16: 164–184. 10.1111/j.1744-6198.1977.tb00632.x 244357

[7] Grady KL , Dracup K , Kennedy G *et al* Team management of patients with heart failure: a statement for healthcare professionals from The Cardiovascular Nursing Council of the American Heart Association. Circulation, 2000; 102: 2443–2456. 11067802 10.1161/01.cir.102.19.2443

[8] Greene J , Hibbard J . Why does patient activation matter? An examination of the relationships between patient activation and health‐related outcomes. Journal of General Internal Medicine, 2012; 27: 520–526. 22127797 10.1007/s11606-011-1931-2 PMC3326094

[9] Stewart M . Towards a global definition of patient centred care — the patient should be the judge of patient centred care. Brtiish Medical Journal, 2001; 322: 444–445. 10.1136/bmj.322.7284.444 PMC1119673 11222407

[10] Stewart M . Effective physician‐patient communcation and health outcomes: a review. Canadian Medical Association Journal, 1995; 152: 1423–1433. 7728691 PMC1337906

[11] Stewart M , Brown J , Donner A *et al* The impact of patient‐centered care on outcomes. Journal of Family Practice, 2000; 49: 796–804. 11032203

[12] Robinson J , Callister L , Berry J , Dearing K . Patient‐centered care and adherence: definitions and applications to improve outcomes. Journal of the American Academy of Nursing Practice, 2008; 20: 600–607. 10.1111/j.1745-7599.2008.00360.x 19120591

[13] Henry BW , McCarthy DM , Nannicelli AP , Seivert NP , Vozenilek JA . Patients' views of teamwork in the emergency department offer insights about team performance. Health Expectations, 2013. doi: 10.1111/hex.12148. 10.1111/hex.12148 PMC5055233 24118891

[14] DeBehnke D , Decker C . The effects of a physician‐nurse patient care team on patient satisfaction in an academic ED. American Journal of Emergency Medicine, 2002; 20: 267–270. 12098169 10.1053/ajem.2002.34199

[15] Triolo P , Hansen P , Kazzaz Y , Chung H , Dobbs S . Improving patient satisfaction through multidisciplinary performance improvement teams. Journal of Nursing Administration, 2002; 32: 448–454. 12360116 10.1097/00005110-200209000-00006

[16] Fokkens AS , Wiegersma PA , van der Meer K , Reijneveld SA . Structured diabetes care leads to differences in organization of care in general practices: the healthcare professional and patient perspective. BMC Health Services Research, 2011; 11: 113–121. 21600064 10.1186/1472-6963-11-113 PMC3116472

[17] Kaasalainen S , Strachan P , Brazil K *et al* Managing palliative care for adults with advanced heart failure. Canadian Journal of Nursing Research, 2011; 43: 38–57. 21977725

[18] Pinnock H , Kendall M , Murray SA *et al* Living and dying with severe chronic obstructive pulmonary disease: multi‐perspective longitudinal qualitative study. BMJ, 2011; doi:10.1136/bmj.d14. 10.1136/bmj.d142 PMC3025692 21262897

[19] Kendall M , Murray S , Carduff E *et al* Use of multiperspective qualitative interviews to understand patients' and carers' beliefs, experiences, and needs. BMJ, 2009. doi:10.1136/bmj.b4122. 10.1136/bmj.b4122 19828645

[20] Hall MJ , Levant S , DeFrances C . Hospitalization for congestive heart failure: United States, 2000‐2010. NCHS Data Brief, 2012; 108: 1–8. 23102190

[21] McAlister FA , Stewart S , Ferrua S , McMurray JJJV . Multidisciplinary strategies for the management of heart failure patients at high risk for admission: a systematic review of randomized trials. Journal of the American College of Cardiology, 2004; 44: 810–819. 15312864 10.1016/j.jacc.2004.05.055

[22] The Heart and Stroke Foundation . Getting to the heart of the matter. Report on the Health of Canadian, 2015 Available: http://www.heartandstroke.com/atf/cf/%7B99452d8b-e7f1-4bd6-a57d-b136ce6c95bf%7D/HSF-2015-HEART-MONTH-REPORT-V2.PDF, accessed 15 July 2015.

[23] Howlett JG . Specialist heart failure clinics must evolve to stay relevant. Canadian Journal of Cardiology, 2014; 30: 276–280. 24565252 10.1016/j.cjca.2013.12.022

[24] Nasstrom L , Idvall E , Stromberg A . Heart failure patients' descriptions of participation in structured home care. Health Expectations, 2015; 18: 1384–1396. doi:10.1111/hex.12120. 23961912 10.1111/hex.12120 PMC5060886

[25] Harkness K , Spaling MA , Currie K , Strachan PH , Clark AM . A systematic review of patient heart failure self‐care strategies. Journal of Cardiovascular Nursing, 2015; 30: 121–135. 24651683 10.1097/JCN.0000000000000118

[26] Clark AM , Spaling M , Harkness K *et al* Determinants of effective heart failure self‐care: a systematic review of patients' and caregivers' perceptions. Heart, 2014; 30: 683–686. 10.1136/heartjnl-2013-304852 24548920

[27] Lingard LA , McDougall A , Schulz V *et al* Understanding palliative care on the heart failure care team: an innovative research methodology. Journal of Pain and Symptom Management, 2013; 45: 901–911. 23017607 10.1016/j.jpainsymman.2012.04.006 PMC5650481

[28] Luborsky MR , Rubinstein RL . Sampling in qualitative research: rationale, issues, and methods. Research on Aging, 1995; 17: 89–113. 22058580 10.1177/0164027595171005 PMC3207270

[29] Morse JM . Sampling in qualitative research In: Lewis‐Beck MS, Bryman A, Liao TF, (eds) The SAGE Encyclopedia of Social Science Research Methods. Thousand Oaks, CA: SAGE Publications, 2004: 993–996.

[30] Uchmanowicz I , Łoboz‐Rudnicka M , Szeląg P , Jankowska‐Polańska B , Łoboz‐Grudzień K . Frailty in heart failure. Current Heart Failure Reports, 2014; 11: 266–273. 24733407 10.1007/s11897-014-0198-4

[31] Afilalo J , Karunananthan S , Eisenberg MJ , Alexander KP , Bergman H . Role of frailty in patients with cardiovascular disease. American Journal of Cardiology, 2009; 103: 1616–1621. 19463525 10.1016/j.amjcard.2009.01.375

[32] Fried LP , Tangen CM , Walston J *et al* Frailty in older adults: evidence for a phenotype. Journal of Gerontolology BIological Sciences and Medical Sciences, 2001; 56: M146–M156. 10.1093/gerona/56.3.m146 11253156

[33] Tait G , Bates J , LaDonna KA *et al* Adaptive practices in heart failure care teams: implications for patient‐centered care in the context of complexity. Journal of Multidisciplinary Healthcare, 2015; 8: 1–12. 26316775 10.2147/JMDH.S85817 PMC4547636

[34] Arnold JMO , Howlett JG , Ducharme A , *et al* Canadian Cardiovascular Society Consensus Conference guidelines on heart failure–2008 update: best practices for the transition of care of heart failure patients, and the recognition, investigation and treatment of cardiomyopathies. Canadian Journal of Cardiology, 2008; 24: 21–40. 18209766 10.1016/s0828-282x(08)70545-2 PMC2631246

[35] Strachan P , Currie K , Harkness K , Spaling M , Clark AM . Context matters in heart failure self‐care: a qualitative systematic review. Journal of Cardiac Failure, 2014; 20: 448–455. 24735549 10.1016/j.cardfail.2014.03.010

[36] Gallagher R , Luttik M‐L , Jaarsma T . Social support and self‐care in heart failure. Journal of Cardiovascular Nursing, 2011; 26: 439–445. 21372734 10.1097/JCN.0b013e31820984e1

[37] Skaperdas E , Tuepker A , Nicolaidis C , Robb JK , Kansagara D , Hickam DH . Congestive heart failure self‐management among US veterans: the role of personal and professional advocates. Patient Education and Counseling, 2014; 95: 371–377. 24666772 10.1016/j.pec.2014.03.002

[38] Knowles S , Combs R , Kirk S , Griffiths M , Patel N , Sanders C . Hidden caring, hidden carers? Exploring the experience of carers for people with long‐term conditions. Health & Social Care in the Community, 2015; 24: 1–15. doi:10.1111/hsc.12207. 25706665 10.1111/hsc.12207 PMC4744729

[39] Clark AM , Davidson P , Currie K , Krimi M , Duncan AS , Thompson DR . Understanding and promoting effective self‐care during heart failure. Current Treatment Options in Cardiovascular Medicine, 2010; 12: 1–9. 20842477 10.1007/s11936-009-0053-1

[40] Falk H , Ekman I , Anderson R , Fu M , Granger B . Older patients' experiences of heart failure‐an integrative literature review. Journal of Nursing Scholarship, 2013; 45: 247–255. 23617442 10.1111/jnu.12025

[41] Bandura A . Toward a psychology of human agency. Perspectives on Psychological Science, 2006; 1: 164–180. 26151469 10.1111/j.1745-6916.2006.00011.x

[42] Edwards A . From the systemic to the relational: relational agency and activity theory In: Sannino A, Daniels H, Gutierrez KD (eds) Learning and Expanding with Activity Theory, 1st edn Cambridge: Cambridge University Press, 2009: 197–211.

[43] Yang V . Activity theory and SLA In: Robinson P (ed.) The Routledge Encyclopedia of Second Language Acquisition. New York/London: Routledge; 2013: 8–10.

[44] Clark NM . Management of chronic disease by patients. Annual Review of Public Health, 2003; 24: 289–313. 10.1146/annurev.publhealth.24.100901.141021 12415147

[45] Kilo CM , Wasson JH . Practice redesign and the patient‐centered medical home: history, promises, and challenges. Health Affairs (Millwood), 2010; 29: 773–778. 10.1377/hlthaff.2010.0012 20439860

[46] Davis K , Schoenbaum SC , Audet AM . A 2020 vision of patient‐centered primary care. Journal of General Internal Medicine, 2005; 20: 953–957. 16191145 10.1111/j.1525-1497.2005.0178.x PMC1490238

[47] Sheridan NF , Kenealy TW , Kidd JD *et al* Patients' engagement in primary care: powerlessness and compounding jeopardy. A qualitative study. Health Expectations, 2015; 18: 32–43. 23033910 10.1111/hex.12006 PMC5060757

[48] Kuper A , Lingard L , Levinson W . Critically appraising qualitative research. British Medical Journal, 2008. doi:10.1136/bmj.a1035. 10.1136/bmj.a1035 18687726

[49] Lincoln YS , Egon GG . Naturalistic Inquiry. Newbury Park, CA: SAGE Publications, 1985.

